# Antioxidants Bioaccessibility and *Lactobacillus salivarius* (CECT 4063) Survival Following the In Vitro Digestion of Vacuum Impregnated Apple Slices: Effect of the Drying Technique, the Addition of Trehalose, and High-Pressure Homogenization

**DOI:** 10.3390/foods10092155

**Published:** 2021-09-12

**Authors:** Cristina Gabriela Burca-Busaga, Noelia Betoret, Lucía Seguí, Jorge García-Hernández, Manuel Hernández, Cristina Barrera

**Affiliations:** 1 Instituto Universitario de Ingeniería de Alimentos para el Desarrollo de la Universitat Politècnica de València, 46022 Valencia, Spain; cribur@posgrado.upv.es (C.G.B.-B.); noebeval@tal.upv.es (N.B.); lusegil@upvnet.upv.es (L.S.); 2 Centro Avanzado de Microbiología de Alimentos de la Universitat Politècnica de València, 46022 Valencia, Spain; jorgarhe@btc.upv.es (J.G.-H.); mhernand@btc.upv.es (M.H.)

**Keywords:** apple structure, *Lactobacillus salivarius* spp. *salivarius* (CECT 4063), trehalose, high pressure homogenization, in vitro digestion, antioxidants bioaccessibility, air-drying, freeze-drying

## Abstract

To benefit the health of consumers, bioactive compounds must reach an adequate concentration at the end of the digestive process. This involves both an effective release from the food matrix where they are contained and a high resistance to exposure to gastrointestinal conditions. Accordingly, this study evaluates the impact of trehalose addition (10% *w/w*) and homogenization (100 MPa), together with the structural changes induced in vacuum impregnated apple slices (VI) by air-drying (AD) and freeze-drying (FD), on *Lactobacillus salivarius* spp. *salivarius* (CECT 4063) survival and the bioaccessibility of antioxidants during in vitro digestion. Vacuum impregnated apple slices conferred maximum protection to the lactobacillus strain during its passage through the gastrointestinal tract, whereas drying with air reduced the final content of the living cells to values below 10 cfu/g. The bioaccessibility of antioxidants also reached the highest values in the VI samples, in which the release of both the total phenols and total flavonoids to the liquid phase increased with in vitro digestion. The addition of trehalose and homogenization at 100 MPa increased the total bioaccessibility of antioxidants in FD and AD apples and the total bioaccessibility of flavonoids in the VI samples. Homogenizing at 100 MPa also increased the survival of *L. salivarius* during in vitro digestion in FD samples.

## 1. Introduction

Health and wellness are trends that have conditioned the development of the food and beverage industry in the last decades [1]. Concern about boosting the immune system as a way to prevent non-communicable diseases and provide protection from pathogenic viral infections has grown considerably since the COVID-19 pandemic hit the world. As a result of the growing inclination of consumers towards preventive healthcare, the global functional food and beverage market size was valued at USD 258.80 billion in 2020 and is projected to double by 2028 [2]. In relation to ingredients, probiotics held the major share, which is mainly attributed to an increasing awareness regarding their prophylactic and therapeutic potential [3].

The functionality of probiotics is mainly based on bacterial interaction with host gut microbiota through a number of actions including increasing the production of vitamins, antioxidants, and short-chain fatty acids, modifying the intestinal microbiota, reducing the intestinal pH, or improving the intestinal barrier selectivity through higher mucin, immunoglobulin A, and defensins production [4]. Probiotics not only protect humans against gastrointestinal pathogens [5], but they may also alleviate gastrointestinal dysbiosis, lower serum cholesterol, ameliorate cancer and lactose intolerance, and prevent allergic and autoimmune disorders [6]. Due to their power to remedy both systematic metabolic diseases and genetic neurodegenerative disorders, probiotics are considered as the twenty-first century panpharmacon [3]. However, in order to achieve these health benefits, probiotics must be consumed regularly and reach the intestine as a viable strain in appropriate quantities (10^8^–10^9^ cfu/day) [7]. Therefore, probiotics used in food formulations must not only survive to the processing and storage stages, but they must also survive the harsh conditions of the gastrointestinal tract, such as the low pH of the stomach, and the digestive enzymes and bile salts of the small intestine [8]. Therefore, various methods have been proposed to enhance the viability of probiotic bacteria, such as strain and food carrier selection, the addition of prebiotics, cell immobilization, and microencapsulation or the induction of cellular stress-tolerance pathways [9].

With regard to the food matrix, research has mainly focused on the role played by its composition, which is often modified by adding ingredients that act as probiotic growth promoters (e.g., sugars, vitamins, minerals, prebiotics) or protectants (e.g., skim milk powder, whey protein, glycerol, lactose, fat, trehalose) [10]. Although dairy foods are the foods with the greatest potential as probiotic carriers, apples have also been demonstrated to be a suitable alternative [11,12] since they contain polyphenols (dihydrochalcones, flavanols, hydroxycinnamates, and flavanol), vitamins, minerals, lipids, peptides, and carbohydrates [13], in addition to fibers (cellulose, hemicellulose, and pectin) [14], that act as prebiotics. Phenolic compounds in apples are also responsible for the health-protecting effects related to apple consumption [15] due to their antioxidant properties and their capacity to neutralize free radicals and to protect cells against oxidative stress [16,17]. Furthermore, as it was recently reported by the authors of [18] for raw and fried tomato puree inoculated with *L. reuteri*, antioxidants may enhance the viability of probiotics during digestion. This makes some unit operations, such as high-pressure homogenization (HPH), particularly relevant to enhance probiotic survival under adverse conditions either in response to induced stress [19,20] or to the higher release of certain bioactives [21].

Denser, more viscous, or complex matrices have been reported to better protect probiotics during digestion, although they also limit their release [10]. However, studies which focus particularly on food structures are scarce and are based on cheese networks, in which it is easier to modify the structure without significantly varying the composition. Changes in the food structure are in most cases promoted by different processing techniques (e.g., boiling, roasting, frying, freezing, air-drying, freeze-drying). In particular, freeze-drying results in high-value products with advanced rehydration properties, a high porosity, and limited shrinkage which, due to the low processing temperatures, preserves their nutrients, color, aroma, and flavors [22]. On the contrary, convective dried products tend to have a low porosity, a high apparent density, poor rehydration properties and, due to high temperatures and long processing times, they lose a large amount of nutrients, flavor and aroma compounds.

For all the aforementioned reasons, this study aims to evaluate the effect that the stabilization of vacuum impregnated apple slices by convective drying and freeze-drying has on *Lactobacillus salivarius* spp. *salivarius* (CECT 4063) survival and on the bioaccessibility of antioxidants (total phenols, total flavonoids, and total ABTS and DPPH scavenging compounds) during an in vitro simulation of gastrointestinal digestion. Simultaneously, information about the impact of trehalose additions to the snack formulation and of the homogenization at 100 MPa of the liquid containing the probiotic are also gathered.

## 2. Materials and Methods

### 2.1. Microbial Strain and Raw Materials

The selection of both the microbial strain and the raw materials was based on our previous research of probiotic apple snacks with the potential ability to treat and prevent *Helicobacter pylori* infection [23,24,25,26]. Therefore, the *Lactobacillus salivarius* spp. *salivarius* CECT 4063 strain was used as a bacterial culture. The lyophilized strain was supplied by the Spanish Type Culture Collection of Paterna (Valencia, Spain). Following the manufacturer’s instructions, the microbial strain was first reactivated in sterile MRS broth (Scharlau Chemie^®^, Barcelona, Spain) at 37 °C for 24 h and then transferred to commercial clementine juice (Hacendado brand), which was used as a probiotic carrier. Finally, apples (var. Granny Smith) cut into 5 mm slices (20 mm internal diameter and 65 mm outer diameter) were used to host the probiotic. The selection of this solid matrix was based on its high homogeneity and porosity compared to other fruits [27].

### 2.2. Sample Preparation

Different food matrices used in this study were manufactured according to the procedure described in Burca-Busaga et al. [26]. On the one hand, vacuum impregnated samples (VI apples) were obtained in a VT 6130M Heraeus Vacutherm Oven (Thermo Scientific) connected to a LVS 210T laboratory vacuum system (Welch Ilmvac™, Fisher Scientific, Madrid, Spain) by the immersion of the apple slices in the corresponding impregnation liquid in a 1:5 (*w/v*) ratio, the application of a vacuum pressure of 50 mbar for 10 min, and the restoration of the atmospheric pressure, which was maintained for another 10 min. On the other hand, air-dried samples (AD apples) were obtained by drying VI apples in a CLW 750 TOP+ tray dryer (Pol-Eko-Aparatura SP.J.) with a cross flow of air at 2 m/s and 40 °C up to a water activity value of 0.35. Finally, freeze-dried samples (FD apples) were obtained by keeping VI apples at −40 °C for 24 h in a Matek CVN-40/105 ultra-freezer and the further sublimation of the frozen water at −45 °C and 0.1 mbar for 24 h in a Telstar Lioalfa-6 freeze-drier.

As the impregnation liquid, commercial clementine juice (Hacendado brand) containing 9.8 g/L of NaHCO_3_ (Sigma-Aldrich, Madrid, Spain) and 5 g/L of yeast extract (Scharlau Chemie^®^, Barcelona, Spain), inoculated with 1.4 ± 0.3 × 10^6^ cfu/mL of *L. salivarius* and then incubated at 37 °C for 24 h was used (liquid 0%_0 MPa). In certain experiments, 100 g/kg of food-grade trehalose from tapioca starch (TREHATM, Cargill Ibérica) was added to the juice prior to inoculation (liquid 10%_0 MPa). In other experiments, the fermented liquid was homogenized at 100 MPa in a laboratory scale high pressure homogenizer (Panda Plus 2000, GEA-Niro Soavi) before it was used as an impregnation liquid (liquid 0%_100 MPa). Based on previous findings [28], homogenization at 100 MPa was not applied to the liquid containing 10% (*w/w*) of trehalose since it significantly reduced (*p*-value < 0.05) the survival of *L. salivarius* following the in vitro digestion of the inoculated clementine juice. Final counts in any of the vacuum impregnation liquids were in the order of 6 ± 2 × 10^8^ cfu/mL.

### 2.3. In Vitro Simulation of Gastrointestinal Digestion

The in vitro simulation of gastrointestinal digestion of the different food matrices was performed in sterile conditions following the protocol described by García-Hernández et al. [18], with some modifications. Each sample was digested three times in duplicate for the determination of both the microbial and the antioxidant properties.

For the oral stage simulation, apple samples were manually cut into pieces of about 2 × 2 × 5 mm^3^ and mixed with human saliva in a ratio 1:1 (*w/v*) and 1:2 (*w/v*), for non-dehydrated (VI apples) and dehydrated samples (both AD and FD apples), respectively. After grinding with a T 25 digital ULTRA-TURRAX^®^ (IKA®-Werke GmbH & Co. KG, Staufen, Germany) at 8500–9500 rpm for 1 min, the simulated oral bolus was mixed in a ratio 1:5 (*w/v*) with a 3 g/L solution of 3200–4500 U/mg pepsin porcine (Sigma-Aldrich, Madrid, Spain) in a sterile saline solution at 0.5% (*w/v*) adjusted to pH 2 with HCl (0.5 N). Following 2 h of constant agitation at 100 rpm and 37 °C on an orbital shaker (Optic Ivymen Systems TM), the resulting simulated chime was mixed in a ratio 1:1.8 (*w/w*) with a 1 g/L solution of 8 × USP porcine pancreatin (Sigma-Aldrich, Madrid, Spain) in a sterile saline solution at 0.5% (*w/v*) adjusted to pH 8 with NaOH (0.1 N). This mixture remained for another 4 h under constant agitation at 100 rpm and 37 °C on an orbital shaker (Optic Ivymen Systems^TM^).

Sampling for the microbial counts was performed at the end of the oral phase at different times along gastric digestion (0, 10, 30, 60, and 120 min) and at different times along intestinal digestion (0, 30, 60, 120, 180, and 240 min). For each time point, 1 mL of the liquid phase was withdrawn from the reaction vessel. Sampling for the antioxidant properties was only performed at the end of both the gastric and the intestinal phases by separating 1 mL aliquots from the corresponding liquid phase.

### 2.4. Analytical Determinations

#### 2.4.1. Moisture Content and Water Activity

The moisture content was obtained from the weight loss undergone by a certain amount of the sample when dried in a vacuum oven (Vaciotem-T, J.P. Selecta) at 60 °C and 200 mbar until a constant weight was reached. The water activity was measured in a CX-2 AquaLab dew point hygrometer (Decagon Devices, Inc., Pullman, WA, USA) at 25 °C.

#### 2.4.2. Microbial Counts

Colony counts were measured by serial decimal dilution in sterile distilled water and plated on MRS Agar (Scharlau Chemie^®^, Barcelona, Spain). Since the microbial growth did not significantly increase under anaerobic conditions, the plates were incubated in aerobiosis at 37 °C for 24 h. The survival of the microbial strain during each stage of in vitro gastrointestinal digestion was then calculated as the ratio between the microbial concentration at the end of the stage and the microbial concentration at the end of the previous one, both referred to the same basis. Likewise, probiotic survival during the entire in vitro gastrointestinal digestion was calculated as the ratio between the microbial concentration in the liquid phase at the end of the intestinal stage and the microbial concentration of the undigested sample. All survival results were expressed as a percentage.

#### 2.4.3. Antioxidant Properties

Measurements in the case of the undigested samples were carried out on the supernatants resulting from the mixing of 2 g of the VI apple (which was reduced to 0.35 g in the case of AD and FD apples) with 20 mL of an 80:20 (*v/v*) methanol in a water solution, dispersing for 2 min at 5000 rpm with a T 25 digital ULTRA-TURRAX^®^ disperser (IKA^®^-Werke GmbH & Co. KG, Staufen, Germany), shaking in the dark for 1 h at 200 rpm on an orbital shaker (Rotabit, J.P. Selecta, Barcelona, Spain) and subsequent centrifugation at 10,000 rpm and 4 °C in a Medifriger-BL-S (J.P. Selecta, Barcelona, Spain) centrifuge. Measurements at the end of both the gastric and the intestinal stage were directly carried out on the resulting liquid phases, which were diluted with distilled water in a 1:5 (*v/v*) ratio for AD and FD apples.

The total phenol content, total flavonoid content, and the overall antioxidant activity of the target samples were spectrophotometrically determined following the procedures described in Burca-Busaga et al. [26].

The total phenol content, which was obtained from the blue color intensity that results after the reaction between the Folin–Ciocalteu reagent (Sigma-Aldrich, Madrid, Spain) and the phenolic compounds present in the sample, was measured at 760 nm and expressed as mg of the gallic acid equivalents per gram of the dried sample (mg GAE/g dw). Specifically, 125 μL of the sample, 125 μL of the Folin–Ciocalteu reagent, and 500 μL of double distilled water were mixed. Following 6 min of reaction in the dark, 1.25 mL of 7.5% (*w/v*) sodium bicarbonate and 1 mL of redistilled water were added and left to react for another 90 min.

The total flavonoid content, which was assessed following the aluminum chloride method, was measured at 368 nm and expressed as mg of the quercetin equivalents per gram of the dried sample (mg QE/g dw). For that purpose, 1.5 mL of sample reacted for 10 min with 1.5 mL of a 2% (*w/v*) solution of aluminum chloride in methanol.

Finally, the antioxidant activity was measured by the DPPH method, which consists of measuring the color change at 515 nm from deep-violet to pale-yellow undergone by radical 1,1-diphenyl-2-picrylhydrazyl when reduced by antioxidants or other radical species, and expressed as mg of the trolox equivalents per gram of the dried sample (mg TE/g dw). In this case, 100 µL of the sample, 900 µL of methanol, and 2000 µL of a 100 mM DPPH solution in methanol (*v/v*) reacted for 30 min in a spectrophotometry cuvette. A blank was used in which the sample was replaced by the same volume of redistilled water.

The bioaccessibility of each compound, defined as the portion that is released from the food matrix into the gastrointestinal tract and thus becomes available for intestinal absorption [17], was obtained as the ratio from its concentration in the liquid phase at the end of the intestinal stage and the concentration in the sample before digestion.

### 2.5. Statistical Analysis

The statistical analysis of the experimental data was performed by means of a one-way or multifactorial ANOVA (confidence level of 95%) carried out with the Statgraphics Centurion XVI tool.

## 3. Results and Discussion

### 3.1. Effect of the Food Matrix and the Vacuum Impregnation Solution on Probiotic Survival during In Vitro Digestion

**Figure 1:**
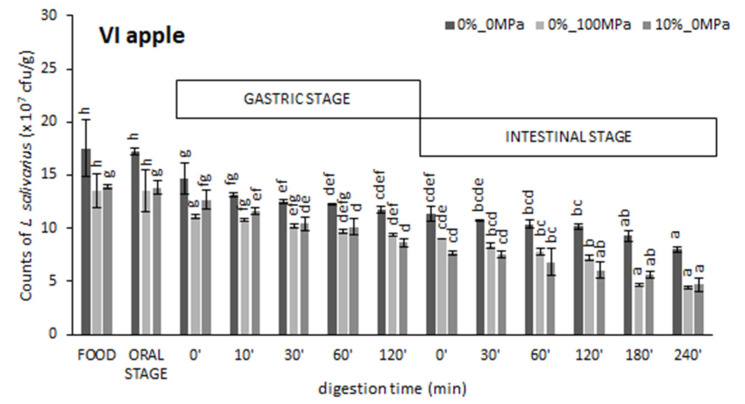

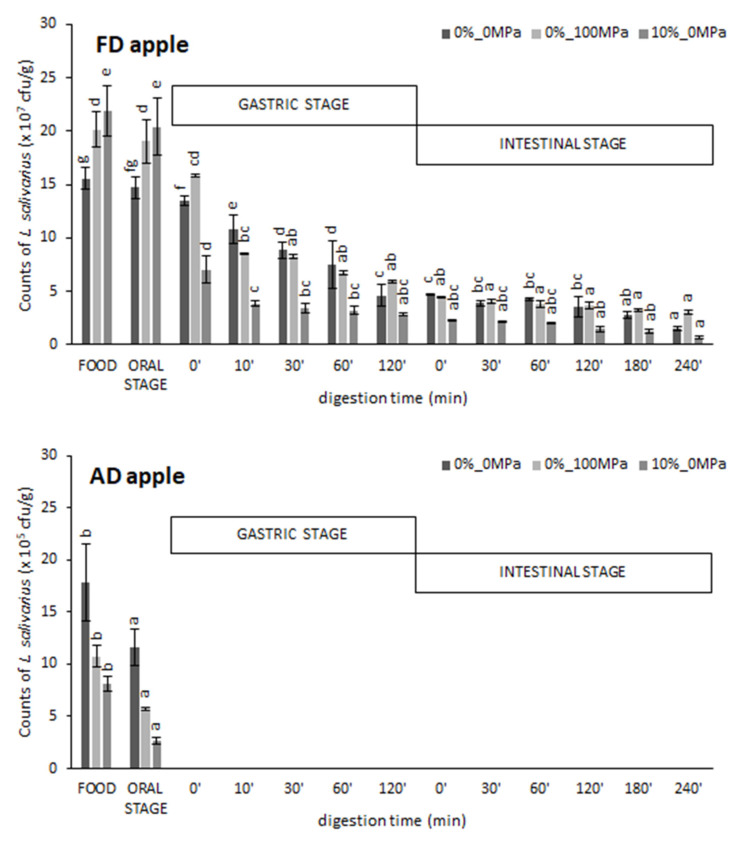
Microbial counts of *L. salivarius* along the gastrointestinal in vitro digestion of different food matrices (VI: vacuum impregnated apples; FD: freeze-dried apples; AD: air-dried apples) made with each of the impregnating solutions. Error bars represent the standard deviation of triplicates from two independent treatments. ^a,b,c,d,e,f,g^ different letters within the same series indicate statistically significant differences (*p*-value < 0.05).

The effect of the food matrix (VI, AD, or FD apples) and the vacuum impregnation liquid (0%_0 MPa, 0%_100 MPa, or 10%_0 MPa) on *L. salivarius* counts along the in vitro simulation of the gastrointestinal human digestion were recorded and are shown in Figure 1. The values obtained for the undigested samples (FOOD) were of the same order as those reported in a previous study by Burca-Busaga et al. [26].

Viable counts in both the VI and FD apples were hardly affected by the exposure to simulated mouth conditions. However, those in the AD apples decreased by 34 ± 4%, 47 ± 6%, and 68.1 ± 1.2% for liquids 0%_0 MPa, 0%_100 MPa, and 10%_0 MPa, respectively. Given that the microbial counts along the in vitro digestion were performed on the liquid phase, it is postulated that the microorganisms were completely released from the solid matrix to the salivary fluid in the VI and FD apples, but only partly in the AD samples. Leaching has previously been reported to be fast and excessive for FD apple cubes and a bit slower for AD apple cubes since freeze-drying causes a more extensive cell wall rupture than the convective process [29]. As for the significantly lower release of *L. salivarius* from the AD apples that were impregnated with the liquid that included 10% of trehalose in its composition, this is consistent with the ability of this disaccharide to replace the water of hydration at the membrane–fluid interface, thus preventing structural collapse as the tissue is dried [30].

A progressive decline in the microbial counts was observed in most cases along the second digestive step. These expected results are attributed to the ability of pepsin to destroy the peptide bond between amino acids and to degrade the microbial cell membrane [31]. However, as pointed out by García-Hernández et al. [18], microbial death due to the shock produced by the gastric juices is more likely to occur in those bacteria whose cell wall had been previously damaged by external factors. In accordance with this, a reduction in the viable number after the gastric stage was minimum in the VI apples (~38 ± 8% on average for samples impregnated with liquids 0%_0 MPa, 0%_100 MPa, and 10%_0 MPa) and increased with the application of a dehydration step (Table 1). Of the two dehydration techniques, freeze-drying was the one with the least impact on the microbial viability (a survival of 21 ± 8% on average for the samples impregnated with liquids 0%_0 MPa, 0%_100 MPa, and 10%_0 MPa). In the case of AD apples, the only contact with the simulated gastric juice reduced the number of viable cells to less than 10 cfu/g. As regards the vacuum impregnation liquid (Table 1), adding 10% of trehalose to its composition slightly reduced the survival of *L. salivarius* during in vitro gastric digestion possibly due to induced osmotic stress. On the contrary, homogenizing the microorganism at 100 MPa had no effect on its survival in VI apples after 120 min of exposure to the simulated gastric juice, but significantly increased that in FD apples. It seems that those cells that survived the freeze-drying step after their homogenization at 100 MPa were better prepared to withstand adverse stomach conditions. In a previous study [28], the survival of *L. salivarius* in gastric simulated conditions after the in vitro digestion of the vacuum impregnation liquids was reported to be 36 ± 7%, 57 ± 3%, and 51.34 ± 0.13% for liquids 0%_0 MPa, 0%_100 MPa, and 10%_0 MPa, respectively. Significantly higher values obtained in the present study after the in vitro digestion of VI apples (63 ± 7% on average) confirms that including the lactobacillus into the porous structure of apple slices protects it as it passes through the upper gastrointestinal tract. In particular, the grip of microorganisms to apples mainly occurs in the intercellular spaces of the parenchymal tissue of the fruit [14]. This finding is particularly interesting in relation to the ability of *L. salivarius* to inhibit pro-inflammatory cytokine secretion from *Helicobacter pylori* (a well-known gastric pathogen) infected cells [32].

Exposure to the small intestinal conditions (pancreatin solution at pH 8.0) resulted in further changes in the survival rate of *L. salivarius*. Again, the loss of viability was significantly higher (*p*-value < 0.05) in FD apples than in VI apples, and while homogenization at 100 MPa slightly increased *L. salivarius* survival in the simulated intestinal juice digestion in FD samples, it significantly reduced it in VI ones (*p*-value < 0.05). Adding 10% of trehalose to the vacuum impregnation liquid negatively affected the survival of lactobacillus during intestinal digestion in both VI and FD apples, but this was much greater in the case of the FD samples in which the disaccharide reached a higher concentration. Given that extracellular trehalose cannot provide sufficient protection for cells during dehydration and gastrointestinal digestion [33], the trehalose added to the growing media was neither imported nor did it induce the expression of trehalose-synthesizing genes to a sufficient extent.

The survival of *L. salivarius* during the entire gastrointestinal digestion was obtained, as explained in Section 2.4.2, from its survival during the simulated oral, gastric, and intestinal stages. As shown in Table 1, the survival of *L. salivarius* in VI apples after the simulated gastrointestinal digestion was similar to that previously reported by Barrera et al. [28] for vacuum impregnation liquids (between 26 ± 5% and 33 ± 2% for liquids 0%_0 MPa and 0%_100 MPa, respectively), but was significantly higher than that obtained for *L. salivarius* in FD apples or in MRS Broth. These differences, however, did not prevent the microorganism from reaching the end of the digestive process in a sufficient concentration (>10^7^ cfu/g) to exert a beneficial effect on the consumer’s health.

The survival of *L. salivarius* during the simulated gastrointestinal digestion of both VI and FD samples was significantly higher (~0.4 log reduction and ~1 log reduction, respectively) than that reported by Valerio et al. (2020) for *L. paracasei* IMPC2.1 in a pectin-coated dehydrated apple snack containing ≥9 log cfu/20 g portion (~2 log reduction). On the same microbial strain, air drying conducted at 60 °C for 1 h, 50 °C for 30 min, and 40 °C up to 24 h was less detrimental to its survival during the digestion process than one-stage drying at 40 °C [25]; on the contrary, the encapsulation of the lactobacillus by HPH at 70 MPa considerably reduced the survival of the strain during the digestion process in FD apple slices.

### 3.2. Effect of the Food Matrix and the Vacuum Impregnation Solution on the Bioaccesibility of Antioxidants during In Vitro Digestion

**Figure 2:**
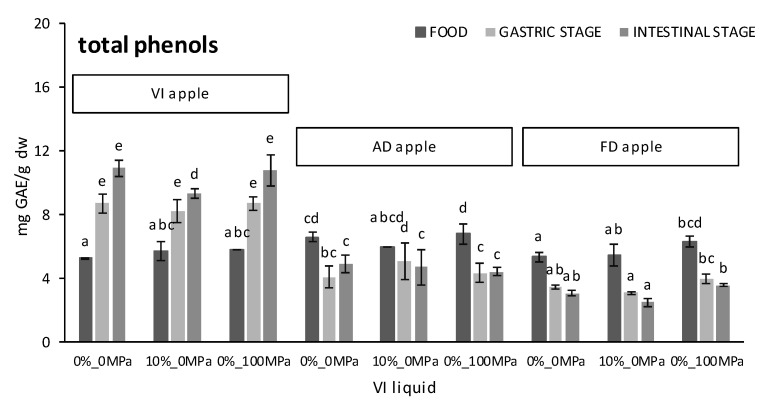

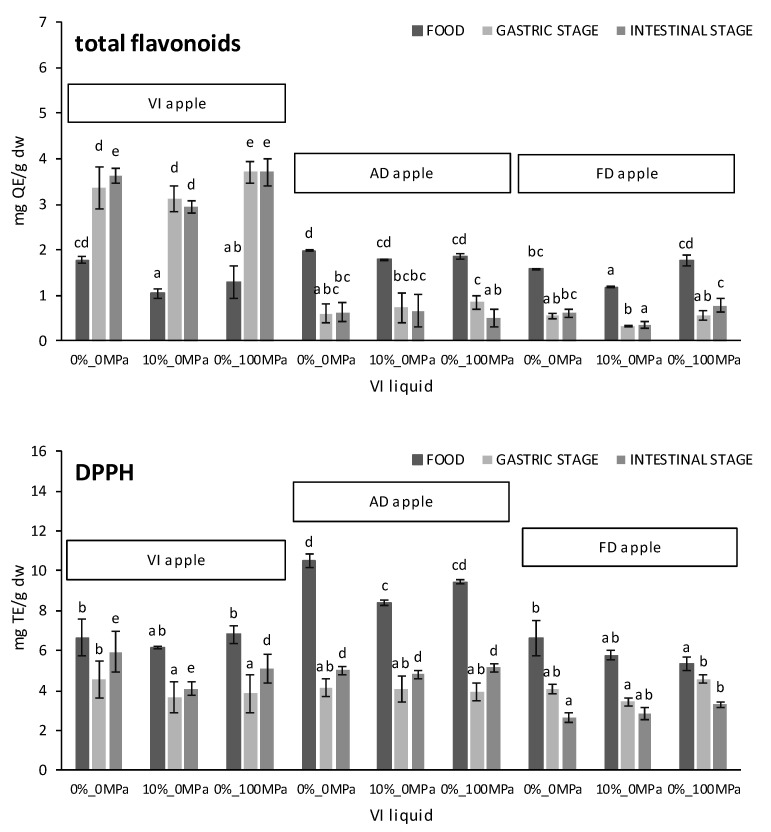
Antioxidant content at each stage of the gastrointestinal in vitro simulation (before digestion and after both the gastric and intestinal stages of digestion) as affected by the food matrix (VI, AD, and FD apples) and the vacuum impregnation liquid (0%_0 MPa, 10%_0 MPa, and 0%_100 MPa). Error bars represent the standard deviation of triplicates from two independent treatments. ^a,b,c,d,e^ different letters within the same series indicate statistically significant differences (*p*-value < 0.05) among samples analyzed at the same moment of the process.

Total phenols (mg GAE/g dw), total flavonoids (mg QE/g dw), and overall antioxidant activity (mg TE/g dw) measured before and after each stage of the in vitro simulation of the gastrointestinal human digestion as affected by the food matrix (VI, AD, or FD apple) and the vacuum impregnation liquid (0%_0 MPa, 0%_100 MPa, or 10%_0 MPa) are shown in Figure 2. In accordance with the previous findings of the research group [26], the values obtained for the undigested vacuum impregnated apples were 5.6 ± 0.4 mg GAE/g dw, 1.4 ± 0.4 mg QE/g dw, and 6.5 ± 0.5 mg TE/G dw, regardless of the composition of the vacuum impregnation liquid used. These values remained almost constant after freeze-drying, but significantly increased (*p*-value < 0.05) after convective drying.

Following the gastric phase of the in vitro digestion, a significant increase (*p*-value < 0.05) in the content of both the total phenols and flavonoids was observed for VI samples. On average, the content of the total phenols and flavonoids increased by 52 ± 11% and 152 ± 37%, respectively, after the action of the simulated gastric juice. This is in agreement with previous findings [34,35] and indicates that the 3 g/L pepsin solution adjusted to pH 2 allowed more glycosidic bonds to be broken and enabled the release of more phenolic compounds (especially of the flavonoid type) than the chemical extraction with an 80% (*v/v*) solution of methanol in water. More specifically, apple samples impregnated with liquid 0%_0 MPa showed the greatest increase in total phenols (from 5.27 ± 0.07 to 8.7 ± 0.6 mg GAE/g dw) but the lowest increase in total flavonoids (from 1.78 ± 0.08 to 3.3 ± 0.5 mg QE/g dw). Unlike the phenolic and flavonoid contents, the DPPH values of the VI apples were 40 ± 4% lower after the gastric phase of digestion, regardless of the composition of the VI liquid. Such a decrease in the antiradical activity could be attributed to the presence of other antioxidant compounds different from polyphenols that are less soluble in the gastric juice and/or more sensitive to the acidic conditions of the stomach. This could be the case with carotenoids which, owing to the numerous double bonds of their chemical structure, are particularly susceptible to oxidation in acidic media [35]. As an example, the overall recovery of carotenoids after the gastric phase of mandarin pulp in vitro digestion was around 79% [36], but it decreased to 36–63% when a blended fruit juice containing orange, pineapple, and kiwi was digested [35].

As to the dried apples, all three antioxidant properties in the samples subjected to gastric conditions were significantly lower (*p*-value < 0.05) than in the undigested ones. A decrease in the total phenols in both AD and FD apples was of the same order (36 ± 4%) and, although in total flavonoids it was slightly higher in FD (68 ± 4%) than in AD apples (59 ± 8%), a reduction in the overall antioxidant activity was significantly higher (*p*-value < 0.05) in AD (57 ± 5%) than in the FD apples (38 ± 15%). Since neither the total content of phenols or flavonoids in the VI apples were negatively affected by the gastric conditions, the decrease observed in both the AD and FD apples could be attributed to the differences in the food matrix between the dry and wet samples. It follows that the release of polyphenols from the solid matrix to the gastric fluid was negatively affected by the structural changes derived from dehydration, regardless of the specific technique used. However, as aforementioned for *L. salivarius*, the leaching of other antioxidant compounds might be more effective from FD samples which are known to suffer a more extensive cell wall rupture. Of all the dehydrated samples, those impregnated with liquid 0%_100 MPa and subsequently FD showed the lowest decline in DPPH values after the gastric stage of the in vitro digestion. This would confirm the application of high-pressure processing on plant foods as a useful tool to improve the extractability and bioaccessibility of antioxidant compounds either through producing changes in the membrane permeability and the disruption of cell walls and cell organelles (as reported by Fernández-Jalao, et al. [17] for “Golden Delicious” apples subjected to 400–600 MPa for 5 min) or through reducing the average particle size (as reported by Di Nunzio et al. [21] for mandarin juice homogenized at 20 MPa).

Following the intestinal phase of the in vitro digestion, the total phenol content of VI apples increased between 15% and 20%, depending on the composition of the vacuum impregnation liquid. However, that of AD and FD apple slices decreased by 11 ± 3%, regardless of the drying technique or the vacuum impregnation liquid used. Based on these findings, it could be said that the total polyphenol release that took place during the gastric digestion of VI apples was uncompleted since vacuum impregnation allows these bioactive compounds to be better retained and protected in the intercellular spaces. This result would be in accordance with that reported by Liu et al. [37], who found that the amount of total extractable phenols released from freeze-dried apple pomace powder increased significantly (from 4.4 to 17.5 mg GAE/g) from the gastric to the jejunal phase due to alkaline hydrolysis causing the breaking of the ester bond linking phenolic acids to the cell wall. However, since Chen et al. [38] also observed that the phenolic content of red delicious apple extracts after the duodenal phase of in vitro digestion was 2.45 times higher than that obtained after the gastric phase, some other changes in polyphenolic compounds such as an interaction with other dietary components, as well as the modification of the chemical structure or solubility might happen. Interestingly, these same changes are considered responsible in other studies for the decrease in the total polyphenols observed in the mild alkaline intestinal conditions [39,40,41]. With regard to chlorogenic acid, the most abundant apple polyphenol, Bouayed et al. [40] reported that between 41% and 77% was degraded during the intestinal digestion of fresh apples, an amount that increased up to 100% in the case of flavanols and caffeic acid belonging to the group of hydroxycinnamic acids. In addition, isomers of chlorogenic acid, mainly cryptochlorogenic acid and neochlorogenic acid, were found to arise in the intestinal phase at concentrations almost comparable to that of residual chlorogenic acid. Nevertheless, the total polyphenolics in the intestinal medium was 40% lower than in the gastric medium. In a different study [41], the total polyphenol content of the air dried apple slices was reported to decrease from 121 ± 21 mg GAE/100 dw to 104 ± 25 mg GAE/100 dw (~14%) due to a pH change from acid (gastric digestion) to alkaline (intestinal digestion), and from 272 ± 8 mg GAE/100 dw to 188 ± 9 mg GAE/100 dw (~31%) when apple slices were enriched with grape juice by vacuum impregnation and ohmic heating at 50 °C before convective drying.

Regarding the total flavonoid content, it remained fairly stable after the intestinal phase simulation and only decreased by 7% on average, regardless of the food matrix and the vacuum impregnation liquid. Out of all the flavonoids in apples, epicatechin, procyanidin B2, and quercetin-3-O-galactoside were found to be the most degraded ones under the weak alkaline conditions of the intestinal digestion [37]. Of the three different matrices analyzed, FD apples were the ones that showed a significantly higher loss of flavonoids (*p*-value < 0.05), while that in VI apples was found to be the lowest. As regards the vacuum impregnation liquid, the 0%_100 MPa was the one that best prevented the degradation of the flavonoids. A similar positive effect on the bioaccessibility of flavonoids as a result of the ortanique low pulp juice homogenization at 20 MPa was previously observed by Di Nunzio et al. [21] and was explained in terms of the reduction in the particle size of the juice that facilitates the release of bioactives from the matrix.

Compared with the gastric phase of digestion, the overall antioxidant activity of both the VI and AD samples increased significantly (*p*-value < 0.05), but particularly in VI apples when liquid 0%_0 MPa was used as an impregnation solution, after the intestinal phase of digestion. On the contrary, the DPPH values of the FD samples decreased by 36 ± 4%, 21 ± 3%, and 28 ± 3% for vacuum impregnation liquids 0%_0 MPa, 0%_100 MPa, and 10%_0 MPa, respectively. An increase in the overall antioxidant activity of VI apples during the intestinal phase was consistent with the increase in the total phenol content. Likewise, a decline in the ability of FD apples to scavenge DPPH radicals matched with the decrease in both the total phenol and flavonoid content. However, the increase in the overall antioxidant activity of AD apples that took place during the intestinal phase despite the decrease in both the total phenol and flavonoid content suggests that novel compounds formed during the hot air-drying processing, such as Maillard-derived melanoidins responsible for browning during the drying process, might still be released to the soluble fraction under alkaline conditions.

**Figure 3:**
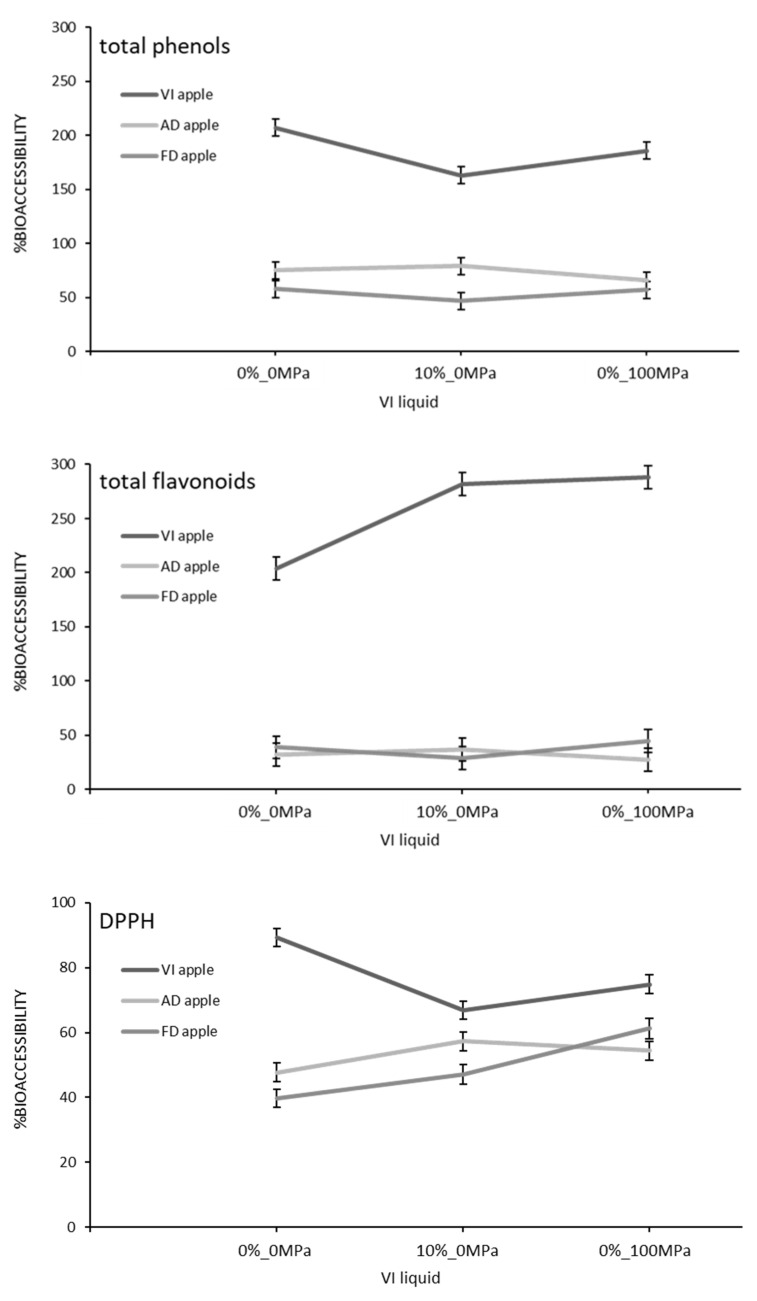
The bioaccessibility of antioxidants as affected by the food matrix (VI, AD, and FD apples) and the vacuum impregnation liquid (0%_0 MPa, 10%_0 MPa, and 0%_100 MPa). Mean values and LSD intervals with a 95% confident level.

All these changes affecting the antioxidant properties of the apple samples along the digestive process have a direct impact on their bioaccessibility, which is a prerequisite for their ability to be effectively absorbed from the intestinal tract into the blood circulation and delivered to the appropriate location within the body [42]. The bioaccessibility of the total phenols, the total flavonoids, and the total antioxidant activity as affected by the food matrix (VI, AD, or FD apples) and the vacuum impregnation liquid (0%_0 MPa, 0%_100 MPa, or 10%_0 MPa) are shown in Figure 3. As it can be observed, the bioaccessibility of the phenolic compounds, including flavonoids, and of all the other antioxidant compounds was significantly higher (*p*-value < 0.05) for VI apples. When liquids 0%_100 MPa or 10%_0 MPa were used as impregnating solutions, the total flavonoids released from VI to the liquid phase at the end of the intestinal stage increased 2.9-fold compared with their content in the undigested samples. This value was reduced to 2.0-fold for vacuum impregnation liquid 0%_0 MPa. A totally opposite effect on the bioaccessibility of both the total phenols and the antioxidants from the VI apples was observed, so that samples impregnated with liquid 0%_0 MPa showed the highest values. In any case, the bioaccesibility values obtained for the VI apples were considerably higher than expected; this could be related to the microbial activity of the lactobacillus strain. This is in line with the findings of Di Nunzio et al. [21], who reported a significant increase in narirutin and didymin bioaccessibility by adding 8 log cfu mL^−1^ of *L. salivarius* to ortanique juice homogenized at 20 MPa. As was also argued by these authors, this result can be attributed to the ability of the microorganism to enhance the release of phenolic compounds linked to fibers and other components of the food matrix. The total phenolic content, anthocyanin, and the DPPH radical scavenging capacity of Sohiong juice were also reported to increase in the presence of *L. plantarum* [43], possibly due to its β-glucosidase activity, as well as to its ability to biotransform the bioactive compounds into their metabolites or to chelate metal ions and to scavenge reactive oxygen species.

The bioaccessibility values obtained for both the AD and FD apples were of the same order: between 46.5% and 78.9% for total phenols, between 27.5% and 44.5% for total flavonoids, and between 39.7 and 61.2% for DPPH scavenging potential. In comparison, Bouayed et al. [40] found that the bioaccessibility of phenolic compounds in apples was 55%, which is in the range of the values obtained in the present study, and Gullon et al. [42] found that the bioaccessibility of polyphenolic compounds present in apple bagasse flour was 91.58%, which is much higher than the values obtained in the present study. Only significant differences (*p*-value < 0.05) were found between AD and FD apples for the bioaccessibility of the total phenols and the total antioxidant values and when liquid 10%_0 MPa was used as the impregnating solution, with AD apples showing higher values than FD ones. Regarding the vacuum impregnation liquid, it hardly affected the bioaccessibility of both the total phenols and flavonoids, but it did affect that of the total antioxidants. In general terms, both the addition of trehalose and the homogenization at 100 MPa increased the bioaccessibility of the total antioxidants in FD and AD apples.

## 4. Conclusions

The study shows that both the survival of *L. salivarius* and the bioaccessibility antioxidants during in vitro digestion were affected by the food matrix of which they are a part. The greatest viability was found when the microorganism was incorporated into the apple’s porous structure by means of the vacuum impregnation technique, but it decreased significantly with the application of a dehydration step. The bioaccessibility of antioxidants also reached the highest values in vacuum impregnated samples, in which both the total phenol and total flavonoid release to the intestinal liquid phase doubled that which was present in the undigested food. The addition of trehalose and the homogenization at 100 MPa increased the bioaccessibility of the total antioxidants in FD and AD apples and the bioaccessibility of the total flavonoids in the VI samples. Homogenizing at 100 MPa also increased the survival of *L. salivarius* during in vitro digestion in the FD samples.

## Figures and Tables

**Table 1:** Microbial concentration of the digested samples (final counts) and the survival percentage of *L. salivarius* to each stage (oral, gastric, and intestinal) and the entire simulated digestion (total) as affected by the food matrix and the growing media. Mean value ± standard deviation of triplicates from two independent treatments.

Treatment		Oral Stage $\frac{X_{OS}^{lb}}{X_{UF}^{lb}}\cdot 100$	Gastric Stage $\frac{X_{GS}^{lb}}{X_{OS}^{lb}}\cdot 100$	Intestinal Stage $\frac{X_{IS}^{lb}}{X_{GS}^{lb}}\cdot 100$	Total $\frac{X_{IS}^{lb}}{X_{UF}^{lb}}\cdot 100$	Final Counts (Log cfu/g)
MRS Broth		-	33 ± 2 ^b^	48.50 ± 0.13 ^bc^	15.8 ± 1.4 ^b^	8.566 ± 0.004 ^f^
VI apple	0%_0 MPa 0%_100 MPa 10%_0 MPa	97 ± 4 ^de^ 100 ± 2 ^e^ 93 ± 7 ^d^	68 ± 3 ^d^ 65 ± 7 ^d^ 55 ± 9 ^c^	68 ± 8 ^d^ 48 ± 2 ^b^ 54 ± 6 ^bc^	47 ± 5 ^d^ 31 ± 4 ^c^ 28 ± 5 ^c^	7.904 ± 0.012 ^e^ 7.644 ± 0.015 ^d^ 7.67 ± 0.06 ^d^
FD apple	0%_0 MPa 0%_100 MPa 10%_0 MPa	99 ± 2 ^de^ 94 ± 4 ^de^ 93 ± 2 ^de^	17 ± 5 ^a^ 28 ± 5 ^b^ 14 ± 3 ^a^	53 ± 9 ^bc^ 58 ± 5 ^c^ 24 ± 3 ^a^	9 ± 2 ^a^ 16 ± 3 ^b^ 3.1 ± 0.2 ^a^	7.19 ± 0.05 ^c^ 7.438 ± 0.012 ^b^ 6.82 ± 0.07 ^a^
AD apple	0%_0 MPa 0%_100 MPa 10%_0 MPa	66 ± 4 ^c^ 53 ± 6 ^b^ 31.9 ± 1.2 ^a^	n d n d n d	n d n d n d	n d n d n d	n d n d n d

^abcde^ different superscripts in the same column indicate statistically significant differences (*p*-value < 0.05). $X_{UF}^{lb}$ are the colony counts of undigested food (cfu/g dw); $X_{OS}^{lb}$ are the colony counts at the end of the oral stage (cfu/g dw); $X_{GS}^{lb}$ are the colony counts at the end of the gastric stage (cfu/g dw); and $X_{IS}^{lb}$ are the colony counts at the end of the intestinal stage (cfu/g dw).

## Data Availability

The data presented in this study are available on request from the corresponding author.
